# Supplementary figures and images for: Nutraceuticals known to promote hair growth do not interfere with the inhibitory action of tamoxifen in MCF7, T47D and BT483 breast cancer cell lines
Source: PLoS One. 2024 Feb 26;19(2):e0297080. doi: 10.1371/journal.pone.0297080 (PMC10896530; doi:10.1371/journal.pone.0297080)

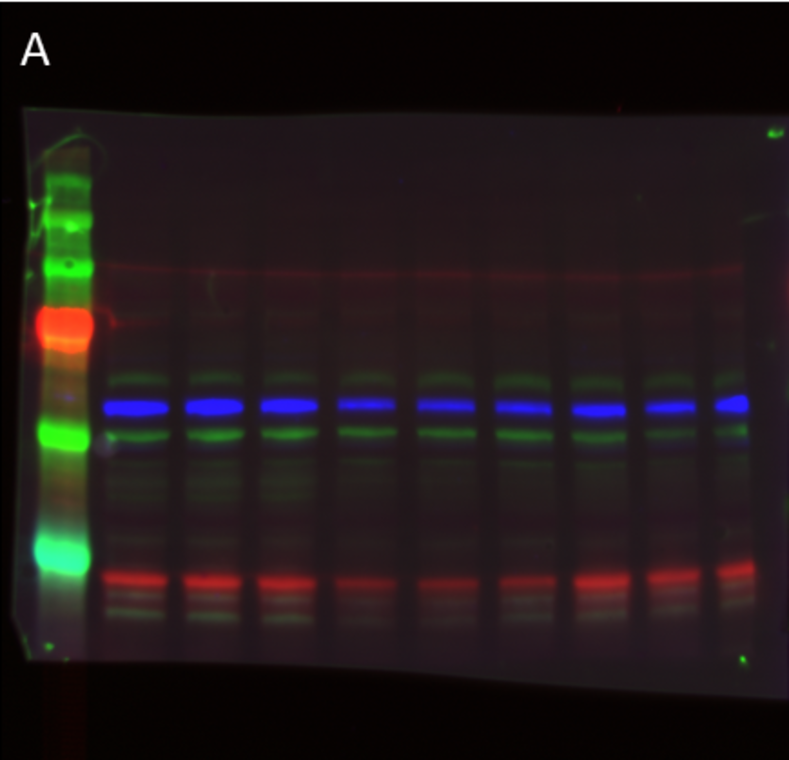

Supplement: S1 Fig — (TIF) [file pone.0297080.s001.tif]
