# Supplementary material for: Nutraceuticals known to promote hair growth do not interfere with the inhibitory action of tamoxifen in MCF7, T47D and BT483 breast cancer cell lines
Source: PLoS One. 2024 Feb 26;19(2):e0297080. doi: 10.1371/journal.pone.0297080 (PMC10896530; doi:10.1371/journal.pone.0297080)
Supplement: S1 Table — 2 log-fold dilutions from the maximum tolerated concentration were also tested in subsequent experiments. (DOCX) [file pone.0297080.s003.docx]

**Supplementary Table 1.**

Table showing suggested maximum experimental concentrations based upon the cytotoxicity observed in the WST-1 assays. 2 log-fold dilutions from the maximum tolerated concentration were also tested in subsequent experiments.

|  | **MCF7** | **T47D** | **BT483** | **High/Medium/Low**  **Experimental Dose**  **(µg/ml)** |
| --- | --- | --- | --- | --- |
| **Extract** | **Max tolerated dose**  **(µg/ml)** | | |  |
| Kelp | 100 | 50 | 100 | **50/5/0.5** |
| Astaxanthin | 50 | 10 | 50 | **10/1/0.1** |
| Saw Palmetto | 50 | 50 | 100 | **50/5/0.5** |
| Tocotrienols | 10 | 10 | 50 | **10/1/0.1** |
| Horsetail | 100 | 100 | 100 | **100/10/1** |
| Maca | 100 | 100 | 100 | **100/10/1** |
| Resveratrol | 10 | 10 | 100 | **10/1/0.1** |
| Curcumin | 1 | 10 | 10 | **1/0.1/0.01** |
| Ashwagandha | 100 | 100 | 100 | **100/10/1** |
| Alternative Curcumin | 10 | 10 | 50 | **10/1/0.1** |
